# Homocysteine, vitamin B12 and folate levels in premature coronary artery disease

**DOI:** 10.1186/1471-2261-6-38

**Published:** 2006-09-26

**Authors:** Saeed Sadeghian, Faramarz Fallahi, Mojtaba Salarifar, Gholamreza Davoodi, Mehran Mahmoodian, Nader Fallah, Soodabeh Darvish, Abbasali Karimi, Tehran Heart Center

**Affiliations:** 1 Assistant Professor of Cardiology, Research Department, Tehran Heart Center, Tehran University of Medical Sciences, Tehran, Iran; 2 Assistant Professor of Cardiology, Shahed University, Tehran, Iran; 3 Assistant Professor of Interventional Cardiology, Tehran Heart Center, Tehran University of Medical Sciences, Tehran, Iran; 4 Researcher, Tehran Heart Center, Tehran University of Medical Sciences, Tehran, Iran; 5 Regular member of board, Department of Biostatistics, Shahed University, Tehran, Iran; 6 Associated Professor of cardiac surgery, Tehran Heart Center, Tehran University of Medical Sciences, Tehran, Iran

## Abstract

**Background:**

Hyperhomocysteinemia is known as an independent risk factor of atherosclerosis, but the probable role of hyperhomocysteinemia in premature Coronary Artery Disease (CAD) is not well studied. The aim of this study was to assess the role of hyperhomocysteinemia, folate and Vitamin B12 deficiency in the development of premature CAD.

**Methods:**

We performed an analytical case-control study on 294 individuals under 45 years (225 males and 69 females) who were admitted for selective coronary angiography to two centers in Tehran.

**Results:**

After considering the exclusion criteria, a total number of 225 individuals were enrolled of which 43.1% had CAD. The mean age of participants was 39.9 +/- 4.3 years (40.1 +/- 4.2 years in males and 39.4 +/- 4.8 years in females). Compared to the control group, the level of homocysteine measured in the plasma of the male participants was significantly high (14.9 +/- 1.2 versus 20.3 +/- 1.9 micromol/lit, P = 0.01). However there was no significant difference in homocysteine level of females with and without CAD (11.8 +/- 1.3 versus 11.5 ± 1.1 micromol/lit, P = 0.87). Mean plasma level of folic acid and vitamin B12 in the study group were 6.3 +/- 0.2 and 282.5 +/- 9.1 respectively. Based on these findings, 10.7% of the study group had folate deficiency while 26.6% had Vitamin B12 deficiency. Logistic regression analysis for evaluating independent CAD risk factors showed hyperhomocysteinemia as an independent risk factor for premature CAD in males (OR = 2.54 0.95% CI 1.23 to 5.22, P = 0.01). Study for the underlying causes of hyperhomocysteinemia showed that male gender and Vitamin B12 deficiency had significant influence on incidence of hyperhomocysteinemia.

**Conclusion:**

We may conclude that hyperhomocysteinemia is an independent risk factor for CAD in young patients (bellow 45 years old) – especially in men -and vitamin B12 deficiency is a preventable cause of hyperhomocysteinemia.

## Background

**Figure 1:**
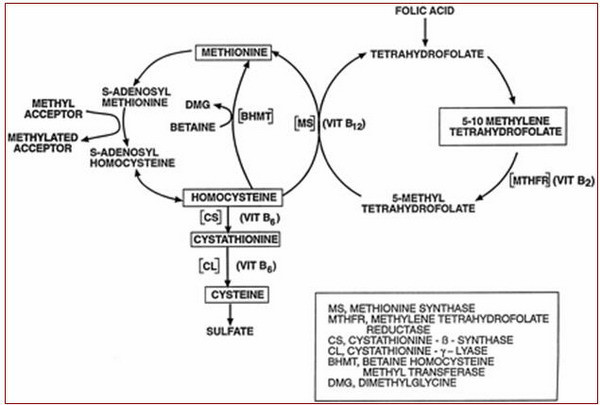
Metabolism of methionine/homocysteine [38].

Homocysteine is a sulfhydryl containing amino acid produced by demethylation of an essential amino acid (methionine) [1]. Methylation of homocysteine, catalyzed by methionine synthetase produces methionine. This enzyme needs vitamin B12 as a co-factor. Homocysteine can also change to cystathionine through the action of the Cystathionine-B-Synthetase (CBS) enzyme [2,4].In humans, vitamin B12 acts as a coenzyme while folic acid provides the methyl essential for the reactions to take place [2,4]. Therefore, folic acid and vitamin B12 deficiency can cause reduction in methylene tetrahydrofolate reductase (MTHFR) activity, leading to decrease in methyonine synthesis and homocysteine accumulation [3,5] (Figure 1).

Except in cases of severe hyperhomocysteinemia (plasma total homocysteine (tHcy) > 100 μmol/lit), usually due to metabolic disorders of methyonine metabolism, mild or moderate cases of hyperhomocysteinemia (tHcy > 15 μmol/lit), prevalent in the general population, can be the result of dietary deficiency of factors such as vitamin B12 and folic acid [5-7]. Other conditions such as polymorphism in the coding gene of MTHFR, consumption of folate antagonists such as carbamazepine and methotrexate and finally disorders of homocysteine metabolism during hypothyroidism and renal failure can also cause hyperhomocysteinemia [8,9]. Recent studies beside demonstrate that diabetes, hypertension, hypercholesterolemia, cigarette smoking and positive family history well known risks in the atherosclerosis phenomena, high plasma level of homocysteine also acts as an independent risk factor of atherosclerosis and coronary artery disease[10-12].

Meanwhile, we do not have sufficient data regarding the role of hyperhomocysteinemia in development of premature CAD and this question needs further investigations [13,14]. Here we should mention that, in most studies, premature CAD is defined as CAD in men under 55 or women less than 65 years old [15]. However, in the Framingham risk stratification table and certain other investigations, the onset of ascending cardiovascular disease in men and women was estimated between the ages of 40–45 years [16]. Based on the above mentioned points, we decided to perform this study in patients less than 45 years in order to have a better evaluation of risk factors in premature CAD. Therefore in the present study, increasing age is not a prominent factor.

In addition to CAD development, high plasma levels of homocysteine have a relation with the incidence of several neurologic disorders such as congenital neural tube defect, Alzheimer's disease and suspicious carcinogenic potentials [17-19].

This study was performed to assess the role of homocysteine, folic acid and vitamin B12 (as probable causes of hyperhomocysteinemia) in the occurrence of premature CAD while considering other known risk factors of CAD.

## Methods

This case-control analytical study was performed in a group of 294 participants (225 male and 69 female) including consecutive patients aged less than 45 years old, who underwent selective coronary angiography into two medical and educational centers in Tehran. From the 294 patients included in this study: 144 were referring angina (56 stable angina and 88 unstable angina); 41 had a previous myocardial infarction; 65 had a positive ergometric test and 44 had a positive scintigraphy test. The sample population was stratified in patients with CAD (127) and normal (167) groups. There were two sources for data collection. Some data were gathered by interview and filling a questionnaire covering all personal data and medical history. This questionnaire was designed based on standard Rose questionnaire for anginal pain, known CAD risk factors and confounding factors.

The second source of data was lab exams [performed for homocysteine, folic acid, vitamin B12, triglyceride, and cholesterol, low density lipoprotein (LDL) and high density lipoprotein (HDL)] and the results of selective coronary angiography. We excluded patients with myocardial infarction in the last 6 weeks, those who had taken any medication containing vitamin B12 or folic acid and N-Acetyl Cysteine (NAC) within 6 weeks of enrollment, those suffering from hypothyroidism ,anemia (hemoglobin<12 g/dl) and creatinine levels equal or greater than 1.5 mg/dl plus any features of chronic renal failure. By applying these criteria, 69 participants (46 males and 23 females) were excluded from the study.

### Laboratory analyses

Blood samples were withdrawn from patients after an overnight fast, and the plasma were put in a cooled container and immediately carried to the "Massoud" laboratory, where the plasma was separated within 1 hour of sampling by centrifugation (20 min, RT, at 2000 RPM) and aliquots were stored at -70°C until analysis. Plasma level of homocysteine was determined by high performance liquid chromatography (HPLC) method, coupled with fluorescence detector and plasma level of folic acid and vitamin B12 by Radio immunoassay (RIA). The serum level of lipids and lipoproteins were determined by calorimetric method. Folic acid levels less than 2.2 ng/ml and vitamin B12 levels less than 175 pg/ml were classified as folate and B12 deficiency, respectively.

### Definitions of risk factors

The traditional or classical coronary risk factors include: (1) diabetes mellitus (positive past history of diabetes and new diabetics, fasting plasma glucose ≥ 120 mg/dl or two hours after glucose load ≥ 200 mg/dl); (2) hypertension (positive past history of hypertension and new hypertensives, systolic blood pressure ≥ 140 mmHg or diastolic blood pressure ≥ 90 mmHg, based on the average of two or more readings on two or more occasions after initial screening); (3) Hyperlipidemia (total cholesterol> 250 mg/dl, LDL > 160 mg/dl or if the patient was on lipid-lowering therapy); (4) smoking (Regularly using tobacco for the last 6 months); and (5) family history of CAD (one or more family member, including parents, brothers and sisters, with documented CAD).

### Statistical analysis

After collection of crude necessary data, these data were statistically analyzed by statistical analysis system (SAS) software for windows 9.1. We used chi-square and t-test to compare qualitative variables and quantitative variables, respectively. Logistic regression analysis was used for detecting independent variables. The differences were considered significant if P value was less than 0.05.

### Ethical consideration

The committee for medical ethics of the Tehran Heart Center approved the study. Each participant signed an informed consent form before completing the questionnaire.

## Results

A total number of 225 participants were enrolled from which 179 (79.6%) were male and 46 (20.4%) were female. All participants were under 45 years old.

Considering results of coronary angiogram, 97 participants (43.1%) had premature coronary artery disease of which 86 patients (88.7%) were male and 11 (11.3%) were female ;128 participants (56.9%) were free of significant CAD of which 93 (72.7%) were male and 35 (27.3%) were female that stratified in control group. In patients with significant CAD; 32 (33%) had single vessel disease, 37 (38.2%) two vessel disease and 28 (28.8%) had three vessel disease.

The mean age of the study group was (40.1 ± 4.2) years in males and (39.4 ± 4.8) in females. No significant difference was noted between the patient and control group in this regard. Major CAD risk factors are compared between controls and patients in Table 1. Significant differences were noted for male gender, diabetes mellitus and hyperlipidemia.

**Table 1:** Characteristics of study population

**Risk factor**	**Case group**	**Control group**	**P value**
Age (year ± SD)	41.01 ± 3.13	39.1 ± 4.94	0.05
Male gender, n (%)	86(88.7)	93(72.6)	0.01
Family history, n (%)	31(31.9)	34(26.5)	0.75
Hypertension, n (%)	24(24.7)	21(16.4)	0.18
Diabetes mellitus, n (%)	13(13.4)	3(0.02)	0.02
Smoking, n (%)	57(58.7)	57(44.5)	0.36
Hyperlipidemia, n (%)	39(40.2)	25(19.5)	0.01

**Laboratory data**			

Triglyceride (mg/dl)	194.68 ± 8.5	177.21 ± 12.4	0.25
Cholesterol (mg/dl)	209.8 ± 4.7	189.34 ± 4.0	0.77
LDL (mg/dl)	132.1 ± 4.1	113.33 ± 2.9	0.16
HDL (mg/dl)	37.7 ± 0.83	41.25 ± 0.92	0.02
Homocysteine (μmol/L)	1.7 ± 19.3	0.9 ± 13.9	0.00

SD, standard deviation; LDL, low density lipoprotein; HDL, high density lipoprotein

The mean serum level of homocysteine was (16.6 ± 1.01 μmol/lit) in the study group and was significantly higher in males (17.9 ± 1.2 μmol/lit) compared to females (11.5 ± 0.9 μmol/lit) (P < 0.01). The mean plasma level of homocysteine in patients (19.3 ± 1.7 μmol/lit) was significantly higher than the control group (13.9 ± 0.9 μmol/lit) (P < 0.005). Considering the sex factor, a similar relationship was present in male participants (P < 0.01) but not in females (P = 0.87) (Table 2).

**Table 2:** Plasma homocysteine level in study population

		**Number (%)**	**homocysteine (μmol/L)**	**P value**
Male	Control	93(52)	14.9 ± 1.2	0.01
	Case	86(48)	20.3 ± 1.9	
Female	Control	35(76)	11.5 ± 1.1	0.87
	Case	11(24)	11.8 ± 1.3	

In order to evaluate the risk of hyperhomocysteinemia for premature CAD in the context of other known CAD risk factors such as diabetes mellitus, hypertension, hyperlipidemia, lipoprotein disorders, positive family history and cigarette smoking, logistic regression test was performed. Results showed that the risk of premature CAD in those participants with a homocysteine plasma level of more than 15 μmol/lit was 2.4 times more than others (OR = 2.42, 95% CI: 1.28–4.56: P = 0.007) (Table 3).

**Table 3:** Multivariate logistic regression analysis for development of coronary artery disease

**Risk factors**	**Odds ratio (OR)**	**Confidence interval 95% (CI)**	**P value**
Male gender	3.05	1.28 – 7.27	0.012
Diabetes mellitus	4.91	1.18 – 20.4	0.029
Hyperlipidemia	2.29	1.16 – 4.53	0.017
HDL	2.05	1.09 – 3.84	0.026
Hyperhomocysteinemia	2.42	1.28 – 4.56	0.007

HDL, high density lipoprotein

Logistic regression test in males with and without hyperhomocysteinemia compared to total subjects showed similar results in effect of hyperhomocysteinemia on development of CAD (OR = 2.54, 95% CI: 1.23–5.22: P = 0.01) while in females there was no increased chance for premature CAD in individuals with hyperhomocysteinemia. However, prevalence of premature CAD was higher in females with hyperhomocysteinemia (above 15 μmol/lit) than who did not have hyperhomocysteinemia (42.9% versus 20.5%). The role of other known risk factors of CAD in increasing the risk of premature CAD is also demonstrated in Table 3.

We studied the relation between hyperhomocysteinemia and plasma level of folic acid, vitamin B12, male gender, increasing age, cigarette smoking, diabetes mellitus and hypertension. Mean plasma level of folic acid in the study group was 6.33 ± 0.29 ng/ml (5.6 ± 0.2 ng/ml in males and 8.8 ± 0.7 ng/ml in females). Mean plasma level of vitamin B12 was 282.5 ± 9.1 pg/ml (283.8 ± 10.5 pg/ml in males and 277.5 ± 18.6 pg/ml in females). Therefore, 10.7% of the study group (24 participants) had folic acid deficiency (13.1% of male participants and 2% of females). Also 24.3% (55 participants) had vitamin B12 deficiency (26.6% of males and 16.3% of females).

Although generally speaking there is a reverse relationship between plasma level of homocysteine and folic acid level (Pearson correlation coefficient = -0.148, P = 0.02) or vitamin B12 (Pearson correlation coefficient = -0.22, P = 0.001), the logistic regression test for the study group showed that male sex (OR = 3.87, 95% CI : 1.57–9.50, P = 0.003) and vitamin B12 deficiency (OR = 2.06, 95% CI : 1.06–3.98 : P < 0.0001) were two factors that caused significant increase in homocysteine levels.

Other factors such as cigarette smoking, increasing age, diabetes mellitus and hypertension did not have any statistically significant role in hyperhomocysteinemia (tHcy>15 μmol/lit). Although the mean plasma level of homocysteine in cigarette smokers (17.5 ± 1.4 μmol/lit) was higher than non-smokers (14.9 ± 1.2 μmol/lit), this was not statistically significant (P = 0.16). Mean plasma level of homocysteine in hypertensive individuals was not significantly different from normotensives (16.2 ± 2.6 μmol/lit versus 16.3 ± 0.9 μmol/lit, P = 0.97). The differences in diabetic and non diabetic individuals were also non significant (13.6 ± 1.4 μmol/lit versus 16.4 ± 1.02 μmol/lit, P = 0.44). No significant difference in plasma homocysteine level was observed in either of age groups (above and bellow 35 years) (P = 0.1).

## Discussion

In the present study, the prevalence of hyperhomocysteinemia in patients with premature CAD was 47.4%. American Heart Association (AHA) reported a prevalence of 12 to 47% in 1999 for patients with CAD [20]. The results of our study show that plasma level of homocysteine in individuals with premature CAD are significantly higher than participants without CAD (19.3 ± 1.7 μmol/lit versus 13.9 ± 0.9 μmol/lit, P = 0.005) and plasma homocysteine levels of more than 15 μmol/lit (hyperhomocysteinemia by definition) were correlated with higher risk of premature CAD. (OR = 2.42, 95% CI: 1.28–4.56, P = 0.007). However, after considering sex as a factor and repeating the statistical tests for subgroups, this conclusion was only valid for male participants. (OR = 2.54, 95% CI: 1.23–5.22; P = 0.01) and there was no increased risk for premature CAD in women with hyperhomocysteinemia. Many studies have shown that elevated total homocysteine concentration is an independent risk factor for cardiovascular diseases. Guo et al in Fokui university (Japan) showed that the plasma level of homocysteine in patients with premature CAD was significantly higher than the control group (15.0 ± 5.7 μmol/lit versus 10.3 ± 5.1 μmol/lit, P < 0.01) [21].

Pinto et al in Bellvitge hospital (Spain) also found a correlation between high plasma level of homocysteine and premature CAD (P < 0.001; OR = 3.2). In their study hyperhomocysteinemia was presented as a risk factor for premature CAD [22].

Lolin et al (China) showed that in patients with premature CAD, plasma levels of homocysteine was significantly higher than normal participants (6.41 μmol/lit versus 5.35 μmol/lit, P = 0.042) [23]. Clarke et al at Adelayde hospital (Ireland) also found quite similar results (OR = 3.2; P = 0.002) [24].

Although, in none of the above mentioned studies was there any difference between males and females in this regard, Foody et al (Cleveland – USA) showed that despite the presence of a correlation between hyperhomocysteinemia and premature CAD in males (OR = 1.93; P = 0.05) no such correlation was noted in females (OR = 1.06; P = 0.89) which resembles our findings [25].

Based on the results of our study and other previous studies it seems that hyperhomocysteinemia is a risk factor for premature CAD in men, but not proven so in women. A Correlation between plasma levels of homocysteine, severity of CAD and number of involved vessels was studied and no significant correlation was found. Brilakis et al have also studied the correlation between plasma level of homocysteine and angiographic findings in patients with CAD and found no significant correlation [26].

Plasma level of homocysteine in male participants was significantly higher than females (17.9 ± 1.2 μmol/lit versus 11.5 ± 5.9 μmol/lit, P = 0.01). Logistic regression analysis yeilded the same results, meaning male sex was an effective factor in plasma level of homocysteine in our study group (OR = 3.87, 95% CI: 1.57–9.5; P = 0.003). Like wise, Jucques et al(Boston- USA) showed that the mean plasma level of homocysteine in men was 11% more than women (P < 0.001). This difference could be having several explanations [27]. First; sex hormones have effects on methionine metabolism; second, the level of creatinine is higher in men; third, the musculoskeletal system of men is usually more developed and has a larger volume and mass compared to women; fourth, plasma level of folic acid and vitamin B12 is different between men and women [28]. But some studies do not confirm these results [29].

In our study, the prevalence of hyperhomocysteinemia was higher than other populations. It could be due to geographical variations, racial and ethnic differences [30], genetic causes, different lifestyle, and inadequate intake of B vitamins and folate, inaccurate cooking of vegetables and not implementing fortification of grain products with folic acid in our country. A low daily intake of vitamin B12 and folic acid could be an eventual cause of hyperhomocysteinemia in our study population. Prolonged cooking of vegetables may destroy up to 90% of folate content [31]. Candidate genes can regulate plasma homocysteine concentrations, especially methylene tetrahydrofolate reductase (MTHFR) and cystathionine-β-synthase (CBS) genes. Recent studies have shown the importance of DNA polymorphisms in the genes for enzymes involved in homocysteine metabolism [32].

Based on results of the present study, the prevalence of folic acid deficiency was 10.7% (13.1% in men and 2% in women) while vitamin B12 deficiency had a rate of 24.4% (26.6% in men and 16.7% in women). This deficiency (especially in vitamin B12) is considerable. Logistic regression tests demonstrate that vitamin B12 deficiency was an effective factor in hyperhomocysteinemia in our study group (OR = 2.06, 95% CI: 1.06–3.98; P < 0.0001) which resembles Jucques et al findings [27]. Despite reports about the role of cigarette smoking, diabetes and hypertension in hyperhomocysteinemia, our study did not show such a relation [33-35]. One of our study limitations is the smaller sample size of female participants in comparison to males. This limitation causes our inability to precisely evaluate the role of hyperhomocysteinemia in young females with CAD.

The present study is one of the few studies in which the control group is chosen precisely and based on angiographic results, while in most of studies, lack of CAD symptoms and signs have been the inclusion criteria for the control group [36,37].

## Conclusion

We may conclude that hyperhomocysteinemia is an independent risk factor for CAD in young patients (bellow 45 years old) (especially in men) and vitamin B12 deficiency is a preventable cause of hyperhomocysteinemia.

## Abbreviations

**AHA: **American Heart Association, **CAD: **Coronary Artery Disease, **CBS: **cystathionine-β-synthase, **HDL: **high density lipoprotein, **HPLC: **High performance liquid chromatography, **LDL: **low density lipoprotein, **MTHFR: **methylene tetrahydrofolate reductase, **NAC: **N-Acetyl Cysteine, **OR: **odds ratio, **RIA: **Radio immunoassay, **SAS: **statistical analysis system, **tHcy: **plasma total homocysteine

## Competing interests

The author(s) declare that they have no competing interests.

## Authors' contributions

1) SS: principle investigator, has made substantial contributions to conception and design of the study

2) FF: performed angiography

3) MS: performed angiography

4) GD: performed angiography and has been involved in drafting

5) MM: has made substantial contributions to data acquisition and has been involved in drafting and interpretation of data

6) NF: data analysis and substantial contribution

7) SD: has been involved in drafting and interpretation of data

8) AK: has given final approval of the version to be published and revising it critically for important intellectual content

Declaration: All authors read and approved the final manuscript

## Pre-publication history

The pre-publication history for this paper can be accessed here:


